# A late Pleistocene dataset of Agulhas Current variability

**DOI:** 10.1038/s41597-020-00689-7

**Published:** 2020-11-11

**Authors:** Margit H. Simon, Martin Ziegler, Stephen Barker, Marcel T. J. van der Meer, Stefan Schouten, Ian R. Hall

**Affiliations:** 1 NORCE Norwegian Research Centre, Bjerknes Centre for Climate Research, Jahnebakken 5, 5007 Bergen, Norway; 2 Centre for Early Sapiens Behaviour (SapienCE), AHKR Institute, University of Bergen, Bergen, Norway; 3 Department of Earth Sciences, Utrecht University, 3584 CD Utrecht, Netherlands; 4 School of Earth and Environmental Sciences, Cardiff University, Cardiff, CF10 3AT United Kingdom; 5 Department of Marine Organic Biogeochemistry, NIOZ Royal Netherlands Institute for Sea Research, Den Burg, Netherlands

**Keywords:** Palaeoclimate, Palaeoceanography

## Abstract

The interocean transfer of thermocline water between the Indian and the Atlantic Oceans known as ‘Agulhas leakage’ is of global significance as it influences the Atlantic Meridional Overturning Circulation (AMOC) on different time scales. Variability in the Agulhas Current regime is key in shaping hydroclimate on the adjacent coastal areas of the African continent today as well as during past climates. However, the lack of long, continuous records from the proximal Agulhas Current region dating beyond the last glacial cycle prevents elucidation of its role in regional and wider global climate changes. This is the first continuous record of hydrographic variability (SST; δ^18^O_sw_) from the Agulhas Current core region spanning the past 270,000 years. The data set is analytical sound and provides a solid age model. As such, it can be used by paleoclimate scientists, archaeologists, and climate modelers to evaluate, for example, linkages between the Agulhas Current system and AMOC dynamics, as well as connections between ocean heat transport and Southern African climate change in the past and its impact on human evolution.

## Abstract (summary)

**Table:** 

Measurement(s)	temperature of sea surface • salinity
Technology Type(s)	gas chromatography of ketone fractions • gas chromatography • Magnesium-to-calcium ratio measurements of planktonic foraminifera • stable isotope analysis • glycerol dialkyl glycerol tetraether (GDGT) analysis • computational modeling technique
Factor Type(s)	temporal measurement
Sample Characteristic - Environment	hydroclimate • ocean
Sample Characteristic - Location	Agulhas Current

Machine-accessible metadata file describing the reported data: 10.6084/m9.figshare.13019678

## Background & Summary

The mass and salt transport through the Indian-Atlantic Ocean Gateway, via the Agulhas leakage, can be considered as a potential controlling factor in the Southern Hemisphere impacting on the North Atlantic salt budget^1,2^. Today Agulhas leakage of ~5–15 Sverdrup (Sv) is one of the dominant sources of the upper branch of the Atlantic Meridional Overturning Circulation (AMOC), connecting the warm route around the southern tip of Africa with the North Atlantic^3,4^. The advection of salt is communicated north within 2–4 decades^5–7^ suggesting a rather fast impact of Agulhas leakage on the AMOC.

The interest of the palaeoclimate community in Agulhas leakage arose from the finding that peak Agulhas leakage occurred during glacial terminations^8^ and plausibly aided the AMOC to shift to its full-strength interglacial mode^9,10^. This hypothesis builds on a variety of records from within the Agulhas leakage pathway, which inferred fluctuations in the strength of Agulhas leakage over the late Pleistocene epoch based on a variety of faunal and geochemical proxy reconstructions^8,11–18^. These findings are further reinforced by a numerical model study^19^ indicating that the strength of the Agulhas leakage varied by ~10 Sv between glacial and interglacial periods.

Fewer studies have concentrated on the Agulhas Current itself. The relationship between the current and the Agulhas leakage is not well understood. Various models have been put forward determining the modern connection between the two. Early studies proposed that the magnitude of Agulhas leakage is thought to depend on the strength and variability of the upstream Agulhas Current and the location of the retroflection^1^. A decoupling of Agulhas Current variability from Agulhas leakage was proposed by Loveday, *et al*.^20^ whereas van Sebille, *et al*.^21^ concluded a weaker Agulhas Current would lead to more Agulhas leakage. On longer timescales a study by Franzese, *et al*.^22^ suggests, based on strontium isotopes in detrital sediments from core sites along the Agulhas Current system, that reduced glacial leakage must be explained by a weaker current. On orbital- to millennial timescales, Simon, *et al*.^23^ concluded, that changes in temperature and salinity in the Agulhas leakage is at least partly the result of variability in the composition in the current itself and can be a poor indicator of the strength of the leakage.

In addition to the importance for global climate dynamics of Agulhas leakage, its variability also significantly impacts the hydroclimate around the southern tip of Africa. Rainfall intensity today in coastal southeast Africa is positively correlated to Sea Surface Temperature (SST) variability in the southwest Indian Ocean and the Agulhas Current regime^24^. Today the Agulhas Current shapes coastal climate^25^, particularly under ridging high-pressure conditions, when low-level onshore flow of moisture from the Agulhas Current region reaches the narrow coastal belt of the Eastern Cape and KwaZulu-Natal provinces where rain falls. How important is the role of the Agulhas Current heat transport in determining hydroclimate conditions in south-eastern Africa across regions and timescales? Increasing the spatio-temporal scales of records representing past variability in the Agulhas Current main flow path is of high importance to the community to attempt to answer these questions. Despite few existing datasets from locations either positioned to monitor changes of the Agulhas Current, where it originates MD96-2048^26^; or slightly outside its main trajectory MD96-2077^27^; a record from the proximal Agulhas Currents main core flow path spanning mutable glacial cycles is still as of yet missing.

**Fig. 1:**
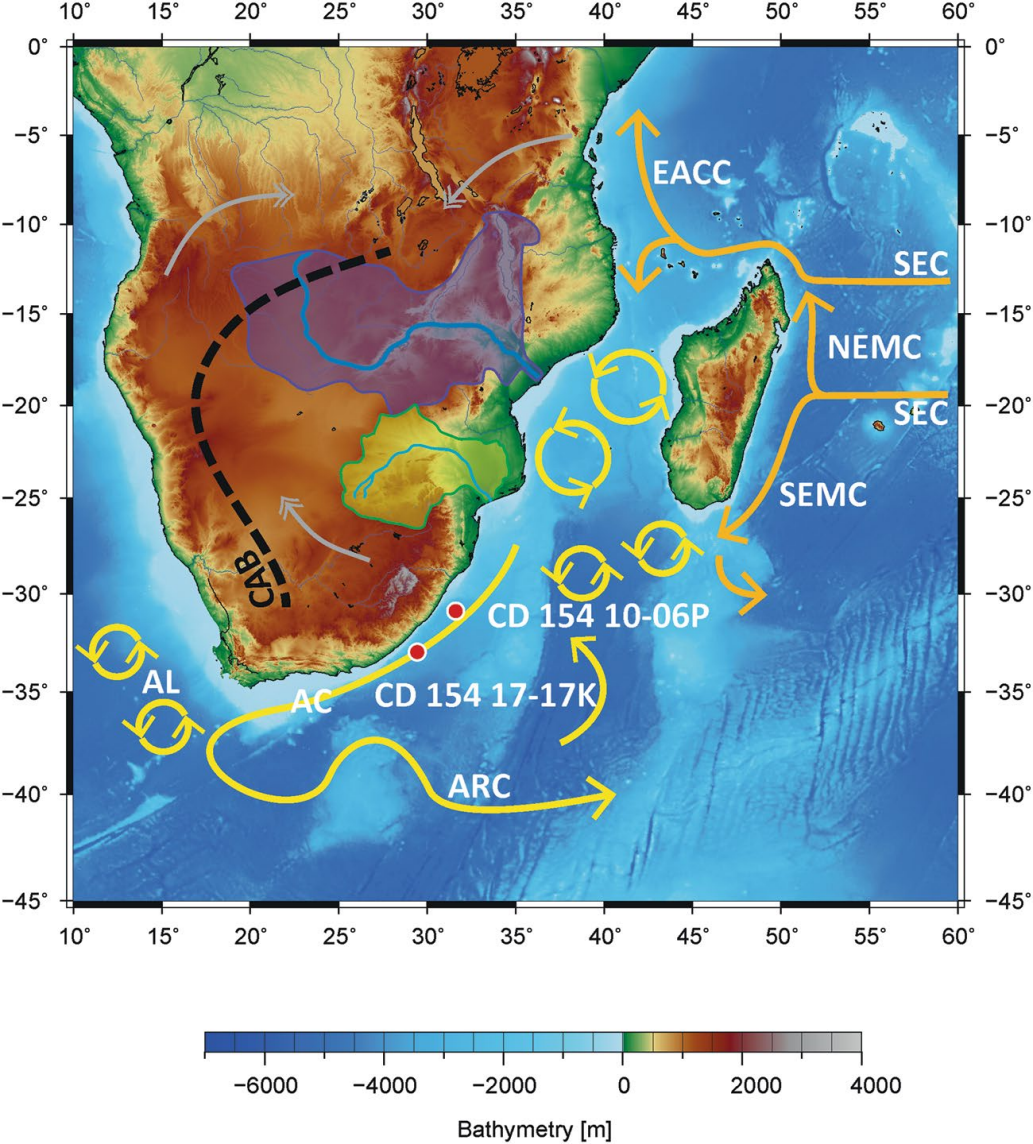
Location map of Site CD154 10-06 P (this study) and CD154 17-17 K^23^ with main surface currents (arrows) in the southwest Indian Ocean and atmospheric circulation over southern Africa during austral summer (December, January, February) with the approximate position of the Congo Air Boundary (CAB) (dashed lines; adapted from Reason *et al*., 2006^77^). AC = Agulhas Current, AL = Agulhas Leakage; SEC = South Equatorial Current, SEMC = South East Madagascar Current, NEMC = North East Madagascar Current, EACC = East Africa Coastal Current, ARC = Agulhas Return Current. Purple shading = Zambezi River Catchment, green shading = Limpopo River Catchment, gray double-headed arrows = main pathways of moisture supply to the African continent from the northwest Atlantic (through Congo) and the northwest and the south-west Indian Ocean. Map Adapted from Hall, *et al*.^78^.

Here we present a new continuous 270-kyr data set consisting of Agulhas Current near-surface temperature and inferred salinity based on surface-dwelling foraminiferal Mg/Ca and δ^18^O records from site CD154 10-06 P (31°10.36’S, 32°08.91’E, 3076 m water depth, Fig. 1). Moreover, we make a multi-proxy compilation of temperature proxies (Mg/Ca; TEX_86_ and U^K’^_37_ based estimates) over the last deglaciation additionally available. This dataset can serve future users to, for example, compare the Agulhas Current SST patterns with South African terrestrial hydroclimate records over the last two glacial cycles to evaluate if the ocean heat transport was shaping coastal land-climate on various timescales. Moreover, the data set can be used to reconstruct the contribution of Agulhas Current water transports to the *T-S* variability in the Indian-Atlantic Ocean gateway and Agulhas leakage over the past 270 kyr. Additionally, the multi-proxy SST compilation over the last deglaciation can serve in future regional data compilations established around South Africa as well as in global temperature compilations.

## Methods

### Age model

**Fig. 2:**
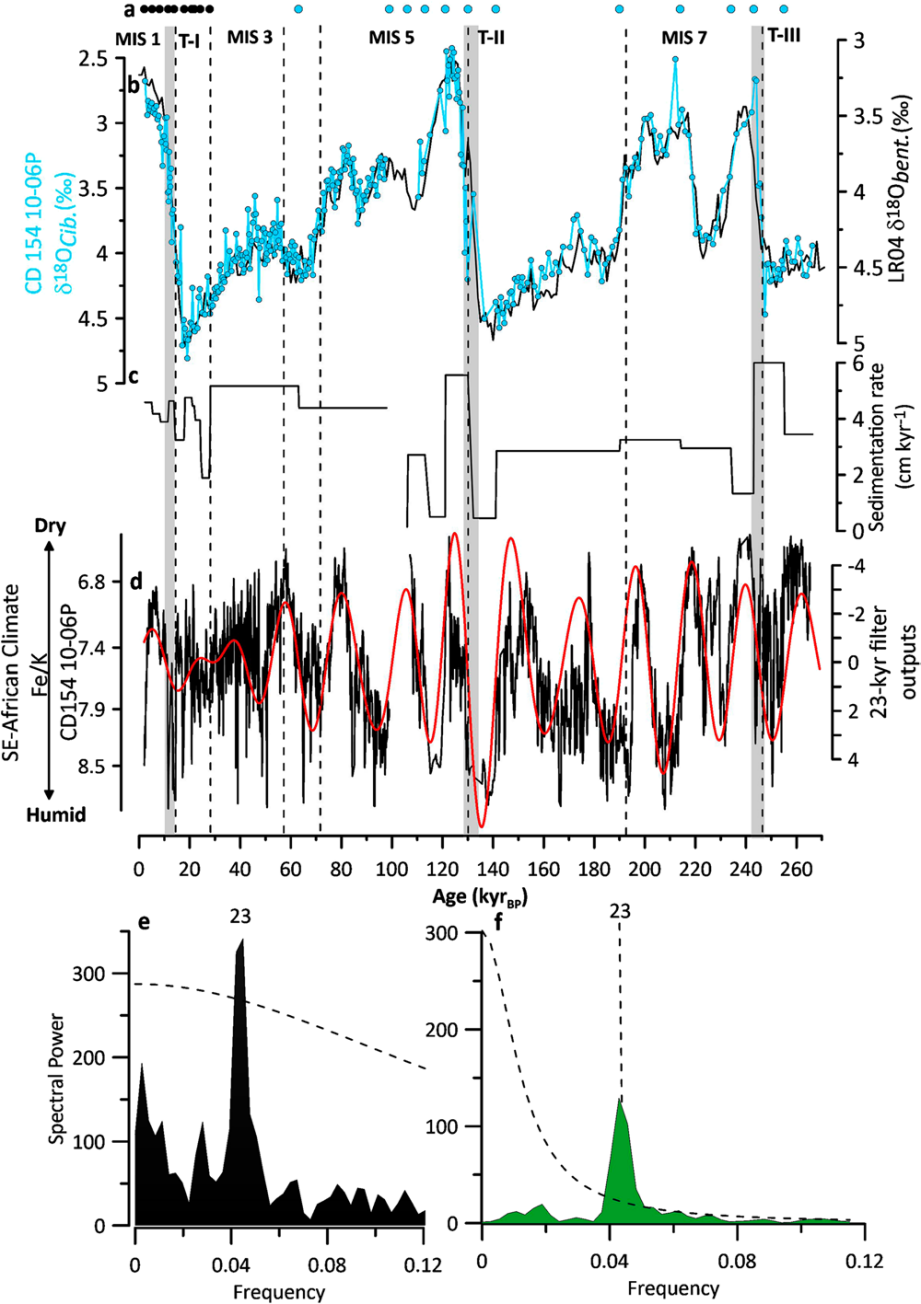
Initial Age model for core CD154 10-06 P. (**a**) Age control points for CD154-10-06P, including radiocarbon dates (black) tuning of the foraminiferal δ^18^O record to LR04 (black) (**b**) Benthic foraminiferal (*Cibicidoides* spp.) δ^18^O record from CD154-10-06P (blue), reflecting global ice volume variability and local deep-water conditions, in comparison with global benthic stack LR04 (black). Marine isotope stages (MIS) are indicated, Underlying grey bars indicate glacial-interglacial Terminations (T) (**c**) Sedimentation rate in cm kyr^-1^ (**d**) Fe/K of CD 154 10-06 P (black, 5 point running mean) with 23-kyr Gaussian filter on top (red) (**e**) Power spectra calculated with the REDFIT-sofware for Fe/K record of core CD154 10-06 P (black) and (**f**) Chinese speleothems δ^18^O record (green), red noise boundaries were estimated as upper 99% chi-squared limits of a fitted AR1 process. Bandwidth is 0.0186. Precession band (23-kyr) is highlighted.

Marine sediment core CD154 10-06 P recovered 969 cm of marine mud mainly composed of foraminiferal ooze (Fig. 2). The core was sampled at 1 cm intervals, the wet sediment was weighed, disaggregated on a rotating wheel for approximately 24 hours, washed over a 63 μm sieve using fine water spray and dried in the oven at 40 °C.

A turbidite was detected during sampling of core CD154 10-06 P through visual inspection and an evident rapid increase in the coarse fraction weight % as well as a sharp drop in the L* record. As such, the identified turbidite interval (50 cm) was removed from the depth scale and the material was not used for the palaeoceanographic records. The age model construction was performed on the new depth adapted scale (for reference please see details in Simon^28^).

The age model for the upper two core sections (upper 100 cm in the core) was developed using ten ^14^C accelerator mass spectrometer (AMS) dates measured from samples containing approximately 1000 tests of the planktonic foraminiferal species *Globigerinoides ruber* (250–315 μm) and has been previously presented in Simon, *et al*.^29^. The age model for the remaining lower core sections was published in detail in Simon, *et al*.^30^. Here we update the radiocarbon ages using the Marine13 calibration curve^31^ with the global mean reservoir correction of (R) 405 years^32^.

**Fig. 3:**
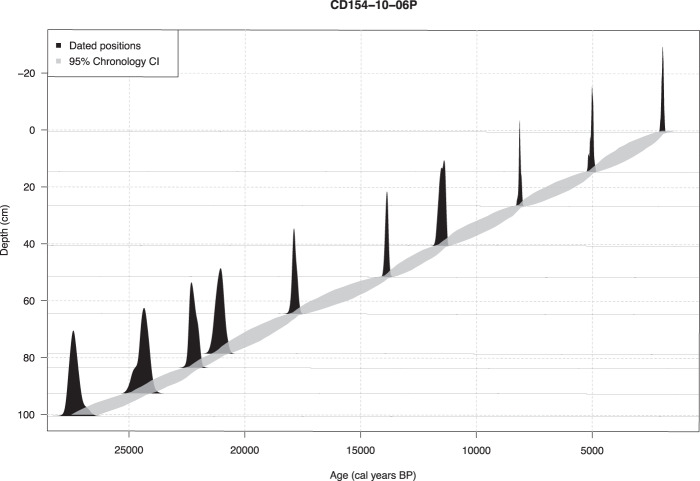
The Bayesian age model obtained by Bchron (black) for the top 100 cm of CD154 10-06 P incorporating a reservoir uncertainty of 405 years (ΔR = 0). Each date is represented by the probability distribution of the intersection between the radiocarbon ages at those depths and the Marine13 calibration curve. The grey shaded area indicates the credible interval (CI) of the 95% probability based on the calibrated dates using the Bayesian statistical package Bchron^34^.

Radiocarbon measurements were made at the Natural Environment Research Council (NERC) Radiocarbon Laboratory (Table 1). The core chronology was constructed using the Bayesian model Bchron^33,34^ from which we derive 95% (2σ) uncertainty on the calibrated ages (Table 1) as well as a 95% probability envelope for each estimated time point (Fig. 3). In the range of the ^14^C dates (1.98–27.38 ka), average sedimentation rates of ~4.0 cm ka^−1^ (1.9–4.8 cm ka^−1^, min and max sedimentation rates, accordingly) and a sample integration of ~300 years for every 1 cm sample is implied. Beyond that, average sedimentation rates are ~4.27 cm ka^−1^ (0.45–6.0 cm ka^−1^, min and max sedimentation rates, accordingly) and an average sample integration of ~1.3 kyr for every 4 cm sample and of ~2.3 kyr for every 8 cm sample is inferred.

**Table 1:** ^14^C dates for sediment core CD154-10-06P.

Depth (cm) CD154 10-06 P	Species	^14^C age BP (yr)	Error +/− 1σ (radiocarbon yrs BP)	2σ credible age interval Lower limit (ka BP)	2σ credible age interval Mid-point (ka BP)	2σ credible age interval Upper limit (ka BP)	Laboratory Code
0–1 cm	*G.ruber*	2359	35	1.895	1.979	2.078	SUERC-45072
14–15 cm	*G.ruber*	4774	35	4.910	5.027	5.195	SUERC-45075
26–27 cm	*G.ruber*	7681	40	8.037	8.141	8.247	SUERC-45076
40–41 cm	*G.ruber*	10409	49	11.296	11.494	11.740	SUERC-45077
51–52 cm	*G.ruber*	12403	63	13.748	13.881	14.020	SUERC-45078
64–65 cm	*G.ruber*	15082	89	17.649	17.865	18.049	SUERC-45079
78–79 cm	*G.ruber*	17863	132	20.735	21.087	21.453	SUERC-45080
83–84 cm	*G.ruber*	18786	148	21.907	22.241	22.484	SUERC-45081
92–93 cm	*G.ruber*	20682	191	23.965	24.380	24.960	SUERC-45082
99–100 cm	*G.ruber*	23498	273	26.758	27.375	27.716	SUERC-45085

Beyond the limits of the radiocarbon method, the visual correlation of the benthic δ^18^O record to the global benthic stack LR04^35^, was used to establish the initial age model (Fig. 2) (Table 2). To further fine-tune the age model, we visually matched common transitions within the Fe/K ratio of core CD154 10–06 P and the δ^18^O splice from Chinese speleothems^36–38^, as presented in Barker, *et al*.^39^ on the precession band (Fig. 2d–f) (Table 2). Ages between each age control point were estimated by linear interpolation. However, it was not possible to establish a continuous age model on the turbidite adapted depth scale (Fig. 2) of the core as the event caused sediment erosion in that interval, which is evident through the absence of half a precession cycle in the Fe/K record during MIS 5 c/d (Fig. 2) (Simon, *et al*.^30^). To adapt for the time gap (~7 kyr), two additional tuning points were used (Fig. 2).

**Table 2:** Age control points for the age model of sediment record CD154-10-06P (*turbidite adaptation).

Radiocarbon dates		Tuning of benthic δ^18^O of CD154-10-06P to LR04^35^		Additional tuning of Fe/K of CD154-10-06P to Chinese speleothem δ^18^O splice	
Depth (cm)	Age (kyr)	Depth (cm)	Age (kyr)	Depth (cm)	Age (kyr)
0.5	1.98	281	63	459	113
14.5	5.03	439*	99	567	153
27.5	8.14	440*	106	602	166
40.5	11.49	463	121	631	178
51.5	13.88	513	130	702	199.5
64.5	17.86	518	141	755	223.5
78.5	21.09	658	190	911	268
83.5	22.41	736	214		
92.5	24.38	795	234		
99.5	27.38				

### Planktonic foraminiferal δ^18^O

Paired stable oxygen isotope (δ^18^O) and Mg/Ca measurements were performed on planktonic foraminiferal species *G. ruber*. Around 60–70 individuals were picked from the 250–315 μm size fraction in core CD154-10-06P for combined analysis. About ¼ of the material was used for stable isotope analysis and ¾ for Mg/Ca measurements after the crushing of shells.

Stable isotopes were measured at Cardiff University, School of Earth and Environmental Sciences using a ThermoFinnigan MAT 253 mass spectrometer linked online to a Carbo Kiel-II carbonate preparation device (long-term external precision is 0.06‰ for δ^18^O and 0.02‰ for δ^13^C). The stable isotope measurements were expressed relative to the Vienna Pee Dee Belemnite scale (VPDB) through calibration with the NBS-19 carbonate standard.

Planktonic foraminifera stable isotopes were analysed every 1 cm in the upper part of the record (Holocene-LGM) and the data set is published in Simon, *et al*.^29^. Further down core samples from 76.5 cm to 152.5 cm yield a 4-cm resolution. From 152.5 cm until the end of the core (918.5 cm) every 8^th^ cm was analysed.

### Mg/Ca Measurements in core CD154-10-06P

Mg/Ca ratios were run every 1 cm in the upper part of the record (Holocene-LGM) and the data set is published in Simon, *et al*.^29^. Further down core from 76.5 cm to 152.5 cm core depth, every 4th cm was analysed. From there on until the end of the core (918.5 cm) every 8^th^ cm was measured.

**Fig. 4:**
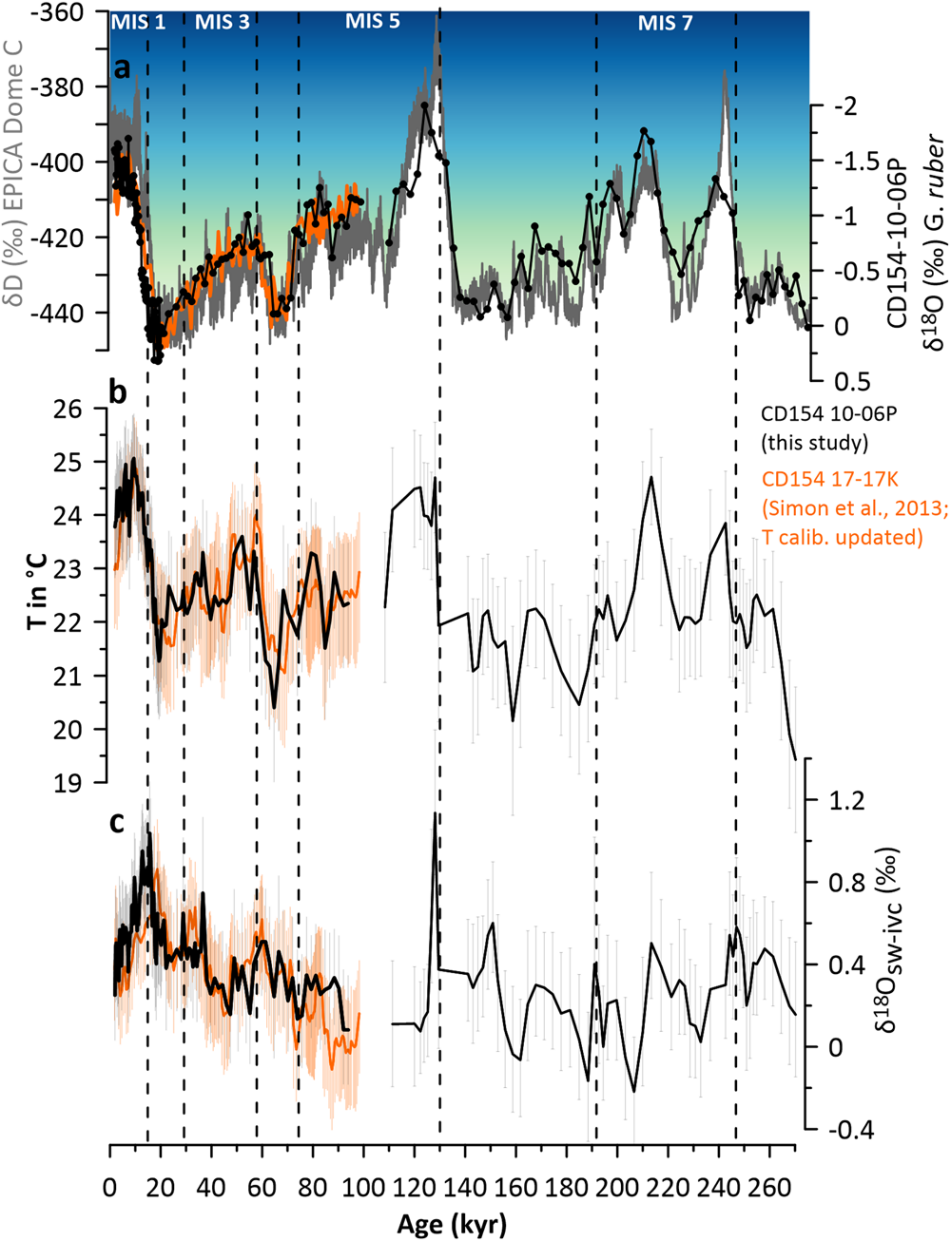
The palaeocenanographic records of core CD154 10-06 P in the main flow path of the Agulhas Current system. (**a**) Comparison of the planktic δ^18^O record (black) of core CD154 10-06 P (black) and upstream site CD154 17-17 K (orange) with the Antarctic European Project for Ice Coring in Antarctica (EPICA) ice-core δD^79^ Antarctica (EPICA) temperature variability as inferred from δD ice record δ^18^O record (**b**) Mg/Ca-based SSTs record of CD154 10-06 P (black) in comparison with upstream site CD154 17-17 K (orange) using PSU Solver output of Mg/Ca-SST-SSS calibration equation based on a compilation of cultured data^42^ with corresponding 2σ confidence intervals (**c**) PSU Solver δ^18^O_sw-ivc_ record indicating inferred relative salinity changes (black) in comparison with upstream site CD154 17-17 K (orange) with corresponding 2σ confidence intervals. Thick black and orange lines are the mean values of the PSU Solver output. Black dashed lines indicate Marine Isotope Stage boundaries.

Magnesium-to-calcium ratio (Mg/Ca) measurements in the planktonic foraminifera *G. ruber* have been used to reconstruct changes in the surface water temperature of the Agulhas Current (Fig. 4). *G. ruber* is a warm water species, highly abundant in the tropical-subtropical waters of the Indian Ocean, and makes up to 40–60% of the planktonic foraminiferal assemblage of the Agulhas Current today^23^. A study of calcification depths of planktonic foraminifera in the tropical Indian Ocean showed that *G. ruber* calcifies within the mixed layer, between 20 and 50 m^40^.

Samples for Mg/Ca analysis were prepared and cleaned following the protocol outlined by Barker, *et al*.^41^. The samples were analyzed using a Thermo Element XR High inductively coupled plasma-mass spectrometry with a long-term precision of element ratios, determined by replicate analyses of standard solutions containing Mg/Ca = 1.15 mmol mol^-1^ and Mg/Ca = 6.9 mmol mol^-1^ of ±1.25% relative standard deviation (RSD) and ±0.52% RSD, respectively. The Mg/Ca ratios of *G. ruber* were converted to calcification temperature using a new Mg/Ca-SST-SSS calibration equation (Mg/Ca = exp (0.084*T + 0.051*S - 2.54)) based on a compilation of cultured data^42^.

### Seawater Oxygen Isotope Reconstruction (δ^18^O_sw_)

We used the computational toolkit Paleo-Seawater Uncertainty Solver (PSU Solver) to derive δ^18^O_sw_ estimates^43^. The Mg/Ca-derived *G. rube*r calcification temperatures were used to determine the oxygen isotopic composition of seawater (δ^18^O_sw_) by extracting the temperature component from the δ^18^O of the calcite using the paleotemperature equation of Bemis, *et al*.^44^ (T = 14.9-4.8 (δ^18^O-δ^18^O_sw_)), with a VPDB to Standard Mean Ocean Water δ^18^O correction of 0.27‰^45^. The δ^18^O_sw_ was corrected for changes in global ice volume to produce ice-volume-corrected local δ^18^O_sw-ivc_ estimates using Spratt and Lisiecki^46^ for global ice volume estimates.

## Organic Proxies

### Sample preparation

Forty sediment samples were freeze-dried and homogenized with a mortar and pestle. The homogenized material was then extracted using an accelerated solvent extractor with dichloromethane (DCM):methanol 9:1 (*v/v*) and a pressure of 1000 psi in 3 extraction cycles. The total lipid extract was separated over an Al_2_O_3_ column into an apolar, ketone and polar fraction using hexane:DCM 9:1, hexane:DCM 1:1 and DCM:methanol 1:1 (v/v), respectively. The ketone fractions were analysed for the alkenone unsaturation index (U^K’^_37_) using a gas chromatograph (GC). The polar fractions were redissolved in hexane/isopropanol (99:1) to a concentration of 2 mg/ml and filtered over a 0.45 PTFE filter and analyzed for the Glycerol dialkyl glycerol tetraether (GDGT) lipid-based TEX_86_ using high-performance liquid chromatography mass spectrometry (HPLC/MS).

### U^K’^_37_ analysis

Ketone fractions were analysed by GC using an Agilent 6890 gas chromatograph with a FID and an Agilent CP Sil-5 fused silica capillary column (50 m × 0.32 mm, film thickness = 0.12 μm) with helium as the carrier gas. The GC-oven was programmed to subsequently increase the temperature from 70 to 130 °C with 20 °C min^−1^ steps, and then with 4 °C min^−1^ steps to 320 °C, at which it was held isothermal for 10 min. The analytical error associated with this method is ±0.2 °C (standard error), U^K’^_37_ values were calculated according to Prahl and Wakeham^47^. Subsequently, SST was calculated using the core top calibration (SST = U^K’^_37_ - 0.044/0.033) established by Müller, *et al*.^48^. The error associated with this calibration is ±1.5 °C on the SST estimates.

### GDGT analysis

Analyses for GDGTs were performed as described by Schouten *et al*.^49^. In summary, an Agilent 1100 series HPLC/MS equipped with an auto-injector and Agilent Chemstation chromatography manager software was used. The separation was achieved on an Alltech Prevail Cyano column (2.1 mm × 150 mm, 3 μm), maintained at 30 °C. GDGTs were eluted with 99% hexane and 1% propanol for 5 min, followed by a linear gradient to 1.8% propanol in 45 min. Flow rate was 0.2 mL min^−1^ by back-flushing hexane/propanol (90:10, *v/v*) at 0.2 mL min^−1^ for 10 min. Detection was achieved using atmospheric pressure positive ion chemical ionization mass spectrometry (APCI-MS) of the eluent. Conditions for the Agilent 1100 APCI-MS 5 were as follows: nebulizer pressure of 60 psi, vaporizer temperature of 400 °C, drying gas (N2) flow of 6 L min^−1^ and temperature 200 °C, capillary voltage of −3 kV and a corona of 5 μA (~3.2 kV). GDGTs were detected by Single Ion Monitoring (SIM) of their [M + H]^+^ ions (dwell time = 234 ms)^49^, and quantified by integration of the peak areas. TEX_86_ was calculated as described by Schouten, *et al*.^50^. The TEX^H^_86_ SST calibration model by Kim *et al*.^51^ (SST^H^ = 68.4*LOG (TEX^H^_86_) + 38.6) was used to transfer TEX_86_ values to absolute SST. This calibration model is recommended for temperature reconstruction above 15 °C^51^ and therefore appears to be the most suitable model for reconstructing subtropical temperatures, as found in the Agulhas Current area. The analytical error associated with this method is ±0.3 °C (standard error)^52^. The error associated with the calibration is ±2.5 °C on the SST estimates^51^.

## Data Records

The here presented data set of marine sediment core CD154 10-06 P was archived using the Linked Paleo Data (LiPD) format^53^. The LiPD framework enables quick querying and extraction, and software in R and Python can help to analyse and visualize paleoclimate data in LiPD format.

Our collection includes data represented in previous publications, but are updated herein, and new data (Table 3). The following table provides an overview:

**Table 3:** Overview of existing datasets and here newly presented proxy records.

Marine sediment core	Proxy/Age model	Timeframe	Publication
CD154-10-06P	δ^18^O, δ^18^O_sw,_ Mg/Ca	1.9-20.3 kyr BP	Simon, *et al*.^29^
CD154-10-06P	TEX_86,_ U^K’^_37_; Mg/Ca-based SST calib. updated	1.9–20.3 kyr BP	**this study**
CD154-10-06P	δ^18^O, δ^18^O_sw,_ Mg/Ca	20.3–270 kyr BP	**this study**
CD154-10-06P	Age model	1.9–20.3; 1.9–270 kyr BP	Simon, *et al*.^29,30^
CD154 17-17 K	δ^18^O, δ^18^O_sw,_ Mg/Ca	1.8–98 kyr BP	Simon, *et al*.^23^
CD154 17-17 K	Age model	1.8–98 kyr BP	Ziegler, *et al*.^80^
CD154 17-17 K	Mg/Ca-based SST calib. updated; δ^18^O_sw_ updated	1.8–98 kyr BP	**this study**

The presented file in the LiPD data format: Agulhas Current_CD154_10-06P.Simon.2020.lpd or in the excel template (Agulhas Current_CD154_10-06P.Simon.2020.xlsx) that can be converted into a LiPD file using the Python LiPD utilities contains a metadata tab that presents the essential marine sediment core information and those of the investigators. Further, the file encloses a tab named paleomeasurement table, which presents all proxy analysis of the data set conducted such as δ^18^O, Mg/Ca ratios, derived δ^18^O_sw-ivc_, calibrated temperatures following a classic approach using a species-specific calibration of Anand, *et al*.^54^, a pH corrected one following Gray and Evans^55^ and the Mg/Ca-temperature-salinity equation after Tierney, *et al*.^42^. Moreover, we present previously unpublished biomarker derived data (Tex_86_ and U^K^’_37_) over the last deglaciation of the same sediment samples.

Radiocarbon dates from the upper part of the core along with calibrated dates, as well as their Bayesian age credible intervals (95%) and downcore age model tie points are provided in the tab: chron measurement tab file.

Notably, the fact that the ^14^C raw data are provided makes the present data set easy to update using a future ^14^C calibration curve. This is also the case for the provided proxy-raw data that can be used to be calibrated and/or corrected in different manners if desired by the user compliant with the recommendations of the Paleoclimate Community reporTing Standard (PaCTS) 1.0^56^

Moreover, we show a published, but updated, dataset upstream in the Agulhas Current (marine sediment core CD154 17-17 K, Figs. 1, 4),^23^ in comparison. The strong correspondence between these two Agulhas Current records testifies that over the last glacial cycle the generated data is reproducible concerning SST and δ^18^O_sw-ivc_ inferred salinity variability, hence, regionally consistent.

All the data sets presented in this study are made available on the SEANOE^57^ database and PANGAEA^58^.

## Technical Validation

### Age model

The age model making use of the alignment of the Fe/K ratio of core CD 154 10-06 P to the δ^18^O splice from Chinese speleothems^36–38^ has been validated by comparing the radiocarbon-dated upper portion of the marine core with the U-Th dated speleothem signal (Fig. 2 in Simon, *et al*.^30^). This validation was the initial step that led to the use of speleothem isotopic records to complement the lower part of the record (Fig. 2). Transferring the speleo-chronology of core CD 154 10-06 P to the record of benthic δ^18^O enables an evaluation of the discrepancies between a ^230^Th-derived chronology and the LR04 isotopic stack which is widely used as tuning target for marine records. The comparison shows that, despite the different tuning approach, a high level of synchronicity between the benthic δ^18^O record of core CD154 10-06 P and the LR04 record is achieved (Pearson T = 0.919; CL 95% (0.86; 0.95)). The average absolute age difference between the initial and the resulting fine-tuned age model is only ~400 years (1σ = 1.47 kyr) verifying our age model approach.

### Quality control and error estimates for Mg/Ca measurements and δ^18^O_sw_ for core CD154 10-06 P

**Fig. 5:**
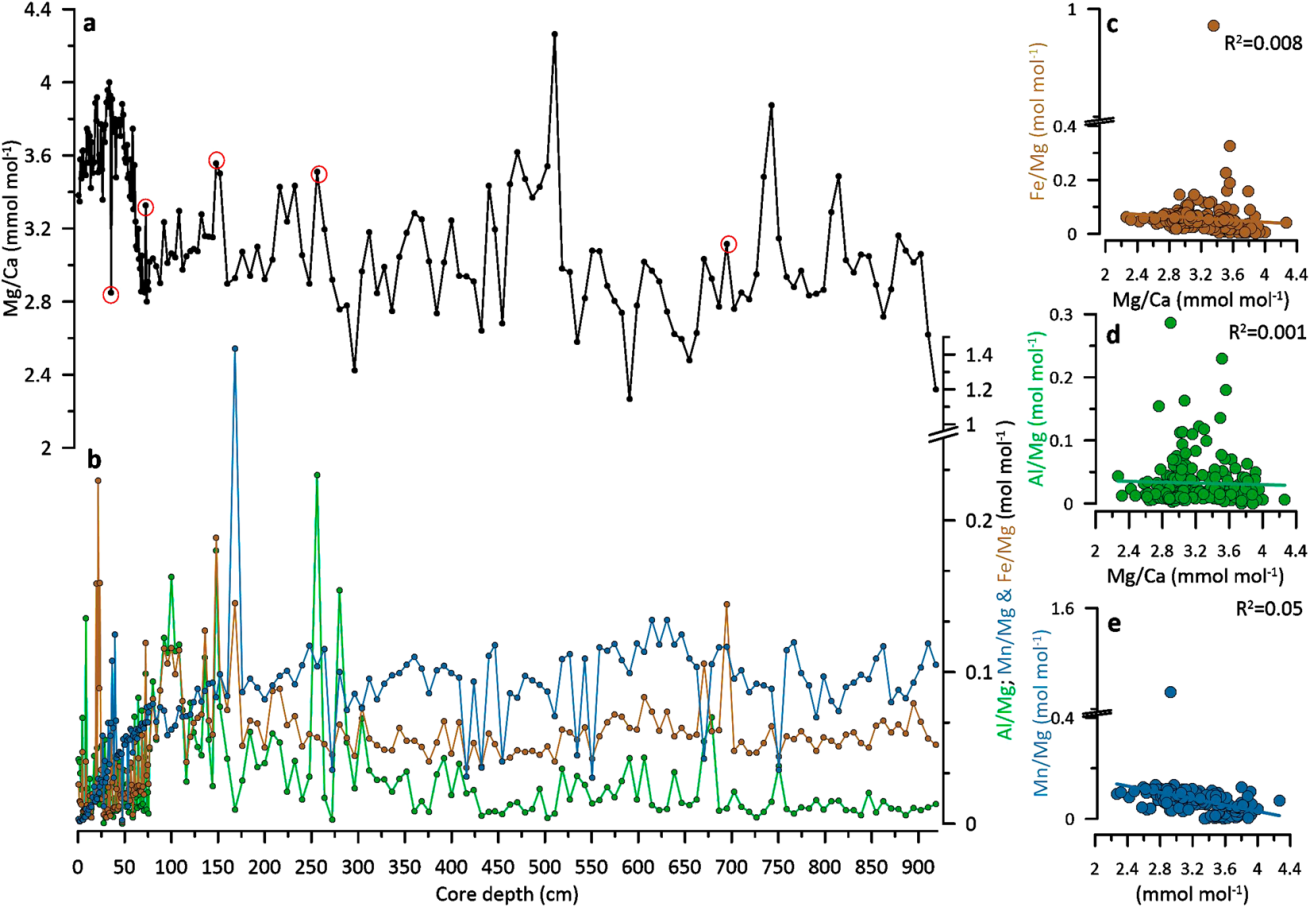
Quality control for Mg/Ca measurements (**a**) *G. ruber* Mg/Ca ratios (mmol mol^−1^) (black circle) (**b**) Fe/Mg (mol mol^−1^) (brown), Al/Mg (mol mol^−1^) (green) and Mn/Mg (mol mol^−1^) (blue) ratios; (**c**) Fe/Mg (mol mol^−1^) (brown) versus Mg/Ca ratios (mmol mol^−1^) and their coefficient of determination R^2^(**d**) Al/Mg (mol mol^−1^) (green) versus Mg/Ca ratios (mmol mol^−1^) and their coefficient of determination R^2^ (**e**) Mn/Mg (mol mol^−1^) (blue) versus Mg/Ca ratios (mmol mol^−1^) and their coefficient of determination R^2^; red circled samples have been removed from the data set.

To validate the analytical robustness of the data set, a screening step consisting to compare Mg/Ca ratios with Fe/Mg, Al/Mg, and Mn/Mg ratios has been undertaken to monitor possible contamination by clays and metal-oxide coatings along the down core results^41,59,60^. Moreover, protocol blanks were routinely run between samples as well. Fe/Mg and Al/ Mg can be used to monitor the potential influence of silicate contamination in foraminiferal Mg/Ca whereas Mn/Mg can determine potential contamination through Mn-Fe oxide coatings^41^. Values above 0.1 mol mol^−1^ for those elemental ratios suggest contamination may be significant. Elevated Fe/Mg ratios (>0.1 mol mol^−1^) are occurring in the core interval 20–27 cm of core CD154 10-06 P but do not seem to co-vary with higher Mg/Ca ratios (Fig. 5.). For that reason, no samples in this core interval have been rejected. One sample (35.5 cm) with particularly low Mg/Ca ratios was rejected as its value falls out of the two-sigma standard deviation range of the entire dataset. In the depth interval 76.5–918.5 cm which is the focus of this study, 3 more samples have been identified and were removed. Those samples were at 148.5 cm displaying higher than average Fe/Mg and Al/Mg ratios; 256.5 cm displaying slightly elevated Al/Mg (0.2 mol mol^−1^) and 694.5 cm that has high Fe/Mg values. Some other intervals display elevated contaminate ratios (>0.1) but do not seem to co-vary with higher Mg/Ca ratios (Fig. 5a).

In order to validate our analytical approach, error estimates are based on the average standard deviation of 0.365 mmol/mol (2σ) accounting for artifacts associated with replication, cleaning procedure, analytical deviations and natural variability of the *G. ruber* populations, which was derived from duplicate measurements of 34 Mg/Ca samples of core CD154 17-17 K^23^. Trace element analyses on material from core CD154 10-06 P were performed in the same manner, on the same species *G. ruber* and analytical facilities as for core CD154 17-17 K. To examine how the influence of salinity on Mg/Ca affect the paleoclimatic reconstructions, we chose a Mg/Ca-temperature-salinity equation^42^. The surface of the modern-day southern Agulhas Current experiences annual temperature variations averaging around 21 °C during winter months and 26 °C during summer months with a mean annual temperature (MAT) of 23 °C (NASA Worldview, 2018, https://worldview.earthdata.nasa.gov). Comparing our derived core top values from the PSU Solver output for SST (23.7 °C; 2σ: 22.70–24.73 °C) with instrumental observations, we find the best match, within error, with MAT with a slight bias towards summer months temperatures.

Overall, to better constrain the various sources of uncertainty that arise from calculating temperature and δ^18^O_sw-ivc_ for our data set (Fig. 4) we use the PSU Solver MATLAB® code that performs bootstrap Monte Carlo simulations to constrain the respective confidence intervals using an iterative approach with user-defined errors, calibrations, and sea-level curves^43^.

**Fig. 6:**
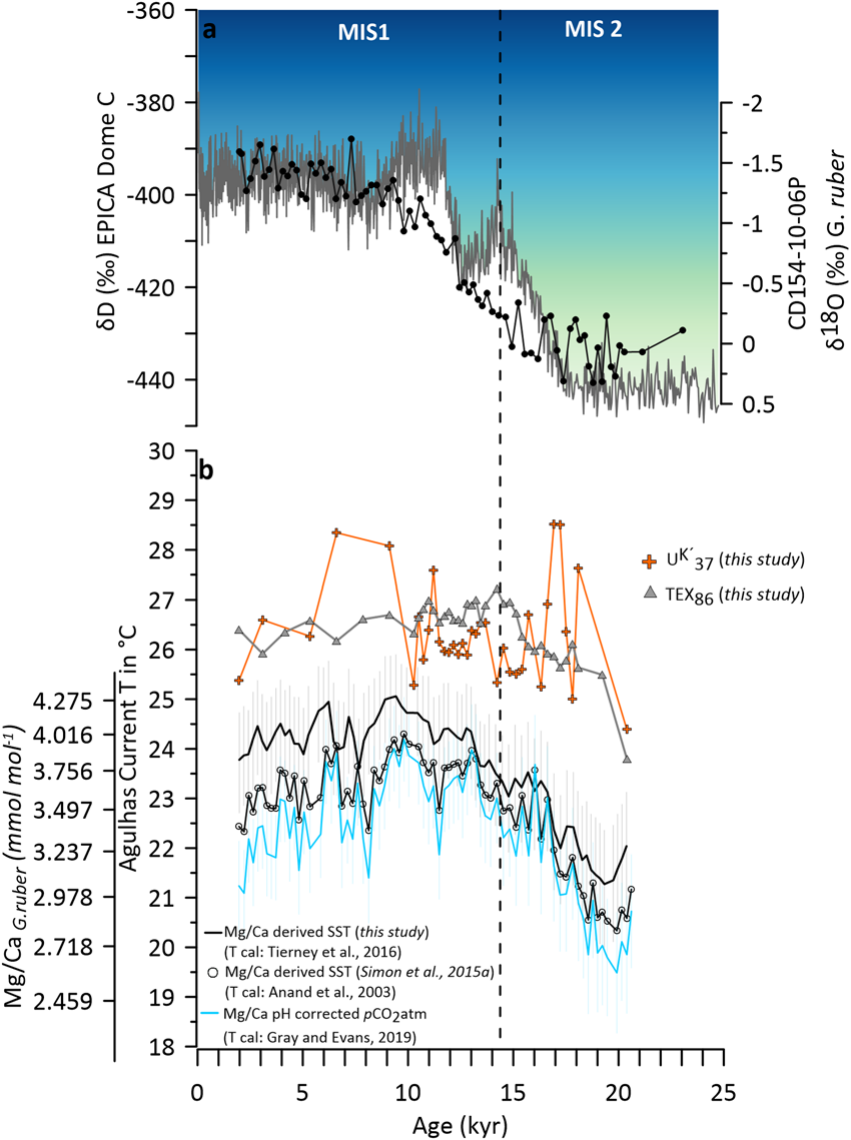
(**a**) Planktic δ^18^O record (black) with the Antarctic European Project for Ice Coring in Antarctica (EPICA) ice-core δD^79^ Antarctica (EPICA) temperature variability as inferred from δD ice record δ^18^O record (**b**) Tex_86_-derived SST record CD 154 10-06 P (grey triangles) reflecting ocean temperature changes in the Agulhas Current, U^K’^_37_-derived SST record (orange crosses) from CD 154 10-06 P; *G. ruber* Mg/Ca ratios (mmol mol^−1^) and derived SST record using^54^, (black circle), derived SST record calculated from Mg/Ca and pH derived from atmospheric pCO_2_ following the species‐specific equation given Gray and Evans^55^; (blue). The error envelopes show the combined 2σ; uncertainty from calibration uncertainty and a salinity uncertainty of ±1 PSU (2σ), derived SST record using Mg/Ca-temperature-salinity equation from Tierney, *et al*.^42^ in black, error envelopes show the combined 2σ range.

Additionally, we demonstrate in Fig. 6 how the choice of different SST calibration curves, as well as proxy choice from the same core material, can influence the final derived SST estimate in a time series spanning from the LGM to the Holocene. Mg/Ca ratios of *G. ruber* converted to calcification temperature using the sediment trap calibration of Anand, *et al*.^54^ (previously published^29^) are shown in comparison with applying a Mg/Ca-temperature-salinity equation^42^ to the same data set. The offsets between both calibrations suggest a significant influence of salinity on Mg/Ca, which alters the structure, and amplitude of change in the resulting reconstruction.

Mg/Ca decreases as pH (and [CO_3_^2−^]) increases, with a sensitivity of ∼5–10% per 0.1 pH unit^61,62^. To validate our data, we used the protocol to correct Mg/Ca for pH down‐core using atmospheric CO_2_ in a new software package “MgCaRB by Gray and Evans^55^, (Fig. 6). The results suggest an only minor influence of pH on our Mg/Ca data set, as the results are almost identical (with a slight cold bias) to the uncorrected results using a species-specific temperature calibration (Fig. 6b). Overall, it should be noted that all outputs for the different Mg/Ca-calibrations agree with each other within error.

However, post-depositional dissolution impacts should also be considered in the future use of the Mg/Ca data. Based on the estimates from Regenberg, *et al*.^63^ water depths below 2 km in the Agulhas Current System are already effected by partial dissolution effects. At the core site CD154 17-17 K, the Δ[CO_3_^−2^] is 0 μmol kg^−1^ and hence below a critical threshold for dissolution of 21.3 ± 6.6 μmol kg^−1^ as suggested by the authors. Taken together, we hence recommend that future users apply calibration equations that include a correction for the dissolution effect on Mg/Ca in foraminiferal calcite such as presented in Dekens, *et al*.^64^ and Regenberg, *et al*.^65^ in addition to the standard calibrations for SST referred to above.

Additionally, U^K’^_37_ and TEX_86_ derived temperature estimates from the same sediment samples are displayed over the last deglaciation (Fig. 6). It can be noted that absolute SST biomarker-based estimates are higher and the pattern over the deglaciation differs from the inorganic based values. The alkenone record yield slightly higher temperatures with larger‐amplitude variations than the archaeal lipid record and both are 2–3 °C higher than the Mg/Ca-based SSTs. The reason for these discordant results among the different paleothermometers might be manifold.

The U^k′^_37_ ratio can be influenced by the ecology of the taxa that produce alkenones^66^. Additionally, alkenones associated with fine‐grained sediment are susceptible to lateral advection in regions of strong currents^67^. The occasionally above the modern seasonal range occurring U^k′^_37_ -based temperatures (>28 °C) in this record might point towards surface drift of material in the Agulhas Current that might originate in the Mozambique Channel where temperature recorded are warmer than at the core site^68^.

We use the global Müller, *et al*.^48^ calibration for U^k′^_37_. Because our site is located in the subtropics, we advise the user to also explore additional estimates produced by e.g. the Bayesian BAYSPLINE approach Tierney and Tingley^69^, which addresses the attenuation of the U^k′^_37_ response to temperature as the U^k′^_37_ ratio approaches one, that is, in locations with SSTs > 24 °C.

There are multiple calibrations for TEX_86_, both global and regional. Empirically, TEX_86_ correlates best to SST or temperatures between 0 and 100 m^51,70^, yet Thaumarchaeota typically reside deeper within the water column. Hence, TEX_86_ may always record subsurface temperatures, and the observed empirical statistical correlation to SST is merely a reflection of the fact that temperatures at ~100 m are highly and significantly correlated with SSTs, at least spatially^71^.

Ho and Laepple^72^ proposed that TEX_86_ reflects purely subsurface ocean conditions and recalibration to deeper depths rather than the surface is required.

Here, we use the global calibrations of Kim, *et al*.^51^ and Kim, *et al*.^70^ and we advise the user to also explore additional estimates produced by e.g. the Bayesian BAYSPAR calibration by Tierney and Tingley^73^ and Ho and Laepple^72^.

### Seawater oxygen isotope reconstruction (δ^18^O_sw_)

To validate the δ^18^O_sw_ reconstructions, a full error propagation performing bootstrap Monte Carlo simulations was conducted accounting for the uncertainty in these reconstructions, which includes age, analytical, calibration, and sampling errors in a framework where the effects of salinity on Mg/Ca, and the effect of ice volume on δ^18^O_sw_ was also incorporated (Fig. 4). The conversion from δ^18^O_sw_ to salinity is generally accompanied by large uncertainties^74^. Therefore, all data is shown here as ice volume corrected δ^18^O_sw_ (δ^18^O_sw-ivc_) values only_._

PSU Solver requires the user to specify a set of input conditions which include (1) the number of total Monte Carlo simulations to be performed, (2) a choice of sea‐level curve (or the option to not correct for ice volume), and (3) the desired set of climate‐geochemistry relationships. Below the transfer functions and error inputs used for the datasets in this study (CD154 10-06 P/ CD154 17-17 K) are listed:

Number of Monte Carlo Simulations mc = 1000:

- δ^18^O_sw_-salinity relationship for the regional southern tropical Indian Ocean (24°S-44°S) of Tiwari, *et al*.^75^: δ^18^O–SSS slope of 0.44 ± 0.03; r^2^ = 0.69; n = 115; significant at p = 0.99
- δ^18^O_c_ paleotemperature equation of Bemis, *et al*.^44^: T = 14.9-4.8(δ^18^O-δ^18^O_sw_)
- Mg/Ca-temperature-salinity equation of Tierney, *et al*.^42^: Mg/Ca = exp (0.084*T + 0.051*S - 2.54)
- sea level estimates: Spratt & Lisiecki *et al*. (2016): 0–798 kyr curve
- analytical uncertainty: δ^18^O_calcite_ and Mg/Ca analysis of G. ruber, respectively: 0.06 permil, 0.365 mmol/mol
- age uncertainty: distributions for radiocarbon ages based on a Bayesian methodology (see Fig. 3 and Table 1 for reference), average age model uncertainty downcore: 1 kyr

Transfer functions and error inputs used in the CD154 17-17 K data set (Fig. 4, orange) were identical, despite that the record of Grant, *et al*.^76^ was used for sea level estimates and an average age model uncertainty of 2.5 kyr was applied downcore.
